# Classifying Unstable and Stable Walking Patterns Using Electroencephalography Signals and Machine Learning Algorithms

**DOI:** 10.3390/s23136005

**Published:** 2023-06-28

**Authors:** Rahul Soangra, Jo Armour Smith, Sivakumar Rajagopal, Sai Viswanth Reddy Yedavalli, Erandumveetil Ramadas Anirudh

**Affiliations:** 1 Fowler School of Engineering, Chapman University, Orange, CA 92866, USA; 2 Crean College of Health and Behavioral Sciences, Chapman University, Orange, CA 92866, USA; josmith@chapman.edu; 3 School of Electronics Engineering, Vellore Institute of Technology, Vellore 632014, India; rsivakumar@vit.ac.in (S.R.); syeda058@uottawa.ca (S.V.R.Y.); erandum@uwindsor.ca (E.R.A.); 4 School of Electrical and Computer Science, University of Ottawa, Ottawa, ON K1N 6N5, Canada; 5 Department of Electrical and Computer Engineering, University of Windsor, Windsor, ON N9B 3P4, Canada

**Keywords:** unstable gait, fall risk, EEG, machine learning, ChronoNet, recurrent neural networks

## Abstract

Analyzing unstable gait patterns from Electroencephalography (EEG) signals is vital to develop real-time brain-computer interface (BCI) systems to prevent falls and associated injuries. This study investigates the feasibility of classification algorithms to detect walking instability utilizing EEG signals. A 64-channel Brain Vision EEG system was used to acquire EEG signals from 13 healthy adults. Participants performed walking trials for four different stable and unstable conditions: (i) normal walking, (ii) normal walking with medial-lateral perturbation (MLP), (iii) normal walking with dual-tasking (Stroop), (iv) normal walking with center of mass visual feedback. Digital biomarkers were extracted using wavelet energy and entropies from the EEG signals. Algorithms like the ChronoNet, SVM, Random Forest, gradient boosting and recurrent neural networks (LSTM) could classify with 67 to 82% accuracy. The classification results show that it is possible to accurately classify different gait patterns (from stable to unstable) using EEG-based digital biomarkers. This study develops various machine-learning-based classification models using EEG datasets with potential applications in detecting unsteady gait neural signals and intervening by preventing falls and injuries.

## 1. Introduction

Falls due to walking instability are common among older adults. About 36 million falls among older adults are reported each year in the United States alone. These falls result in over 32,000 deaths annually, and about 3 million older adults require emergency department treatment for fall-related injuries [1]. One in five falls results in an injury, such as broken bones or head trauma. Hip fractures after falls are a particularly concerning injury, with at least 300,000 older adults hospitalized for hip fractures each year [2,3]. More than 95% of hip fractures are caused by falling, usually sideways, and women are at a higher risk, accounting for three-quarters of all hip fractures [2,3]. It is important to identify instability among older adults and intervene to prevent falls to reduce the risk of injury and death. As people age, their gait, or the way they walk, can become less stable due to a variety of factors such as muscle weakness, changes in balance, and reduced visual acuity. These changes can make it more difficult for older adults to maintain their balance and avoid falls. Improving gait stability through exercise, physical therapy, and other interventions can help reduce the risk of falls and fall-related injuries. It is important for healthcare providers to assess gait stability among older adults and develop individualized plans to improve gait stability and reduce the risk of falls.

Among older adults, walking instability is a major contributor to falls [4,5]. Stability refers to a motor system’s ability to maintain or recover its initial condition in the presence of internal (e.g., neuromuscular) and external (e.g., environmental) disturbances [6,7]. Measures of stability provide important information about the intrinsic noise in motor task performance and directly quantify dynamic error correction performance [8,9]. Brain signal measures may indirectly quantify a person’s gait stability by accounting for inherent noise in the neuromuscular task or the environment, which can bring the individual’s dynamic state closer to their stability limits [6,7].

Recent developments in mobile EEG technologies enable researchers to design experiments describing behavioral and neural responses of walking. In addition, machine learning (ML) algorithms have the potential to automate EEG analysis to improve walking stability. For instance, brain-computer interfaces (BCIs) are devices that analyze EEG signals and translate them into commands that control various devices, such as prosthetic limbs, exoskeletons, computers, and digital avatars. BCIs work by detecting patterns in brain activity that correspond to specific movements or commands. For example, if a person is unstable during walking, certain areas of their brain will become active, generating specific patterns of electrical activity that can be detected by a BCI. These patterns can then be used to control an assistive device, allowing the person to gain stability during walking. BCI systems based on EEG data can be designed for assistive and therapeutical applications for patients who face gait instability and further facilitate rehabilitation among fall prone older adults.

Thus, developing ML models for EEG signals are important since it allows for: (i) salient feature identification related to gait stability, (ii) understanding of underlying neural correlates (or digital biomarkers) of gait stability, and (iii) improved usability for BCI systems. It is often difficult to identify and quantify EEG features related to gait stability since gait is a complex dynamic activity. EEG has contributed to the identification and quantification of important cortical features associated with stable gait. The underlying neural mechanisms involved in unstable versus stable gait are not completely understood but thought to rely on cortical and subcortical centers [8]. Other devices such as fMRI (functional MRI) are capable of unraveling neural correlates of gait [9,10,11,12,13], but do not allow participants to walk. Near infrared spectroscopy (NIRS) is somewhat limited in spatial and temporal resolution [14]. Previous studies with imagined gait using fMRI and NIRS suggest brain activity in subcortical [11,15] and cortical areas [9,12,13,14]. A study by Sipp et al. revealed that stabilized walking on a balance beam may lead to significantly reduced power in the beta frequency band in left and right sensory motor cortex, as well as an increase in theta power in or near anterior cingulate, anterior parietal, superior dorsolateral-prefrontal, and medial sensorimotor cortex [16]. Higher cortical centers are involved to maintain balance in the medio-lateral direction [10,16]. Thus, these areas may be involved in maintaining gait stability.

Classification of human walking stability is highly significant for successful fall reduction. In an EEG/BCI-based rehabilitation system, the brain signals can be non-invasively extracted, processed, and translated for intervention. For an effective fall prevention, it is critical to detect the instability as early as possible to intervene appropriately. The main goal of this study is to provide a basis for a future system capable of classifying gait stability in real-time during ambulation. In this study, we asked participants to walk in four stable and unstable conditions: (i) normal walking, (ii) normal walking with a cognitive task, (iii) normal walking with center of mass (COM) visual feedback, and (iv) normal walking with medio-lateral perturbations. We used state-of-the-art ML methods such as Support Vector Machines (SVM), Random Forest, XG Boosting, Recurrent Neural Network (RNN) and ChronoNet for gait type and stability classifications.

## 2. Materials and Methods

Subjects: A total of 13 healthy subjects (age range 18–35 years, height 157–162 cm, and weight 68–110 Kg, 6 males and 7 females) were recruited in this study. All participants signed the written consent form approved by Chapman University (CU IRB # 20-62). Once the subject had signed the consent form, we proceeded to prepare the EEG cap and electrodes.

Subject Preparation: The participants wore an EEG cap with 64 electrodes (actiCAP, Brain Products GmbH) to record their brain activity. To ensure good electrode–skin contact, gel (SonoGel, Bad Camberg, Germany) was applied to keep impedance below 20 kOhm. The EEG montage was set up according to the 10–20 standard, and both reference and ground electrodes were used to facilitate the post-hoc removal of muscle activity from the EEG. In addition to the EEG setup, 59 reflective markers were placed along the subject’s body landmarks for tracking of segment trajectories, and a safety harness was used to secure participants during all walking trials. The kinematic data were processed using MATLAB (MATLAB 2022a. Natick, MA, USA: The MathWorks Inc.).

**Figure 1:**
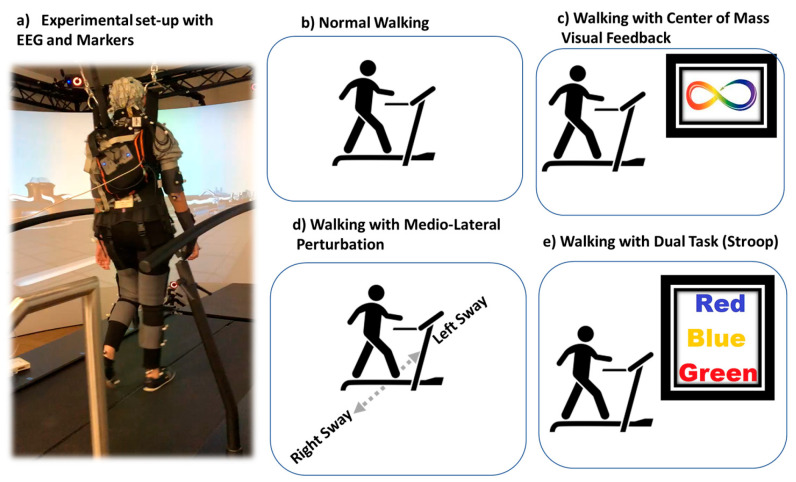
The representative picture of experimental set-up with participant walking on GRAIL treadmill in virtual reality environment is shown in (**a**) and various conditions of walking performed (**b**) normal walking, (**c**) walking with center of mass visual feedback, (**d**) walking with medio-lateral perturbation, and (**e**) walking with dual-tasking (Stroop).

Testing conditions: This study involved walking under four different conditions (Figure 1): normal walking, Stroop, COM visual feedback, and Medio-lateral Perturbation (MLP). The participants conducted trials of each condition for 5 min and were randomly assigned to that condition. A rest of 5 min was provided between each trial for the participants to avoid muscle fatigue and its associated gait changes. During the incongruent Stroop test, the subject was shown a word and asked to say the color of the word displayed. The COM test aimed to evaluate the subject’s ability to maintain their center of mass within a specified boundary. The boundary and center of mass objects were created using the four hip markers, and the subject received real-time feedback during the trial. The normal walking test consisted of just walking without any other conditions and was used as a baseline for the other tests. The MLP test was similar to the walking trial, but the treadmill moved in the medio-lateral direction.

Preprocessing of synchronized EEG signals: The BrainVision Recorder software (BrainVision Recorder, Vers. 1.23.0001, Brian Products GmbH, Gilching, Germany) was used to record the EEG signals, which were then saved in vhdr files and imported into Matlab using EEGLab (Delorme and Makeig, 2004). EEG signals are typically categorized into five sub-bands: alpha, beta, theta, delta, and gamma waves. The delta frequency range, which is the slowest frequency band, starts from 0.5 to 4 Hz, while gamma waves can reach up to 70 Hz. To ensure that the EEG signal was within the range of 0.5–70 Hz, a filtering technique was employed to remove outlier noise. Specifically, a band-pass filter was used with a high-pass threshold of 1 Hz and a low-pass threshold of 70 Hz. Additionally, a band-stop filter ranging from 55 Hz to 65 Hz was utilized to remove electrical noise. All EEG signal processing and classification algorithms were implemented offline. The EEG signals were truncated for each gait cycle and features were extracted.

**Figure 2:**
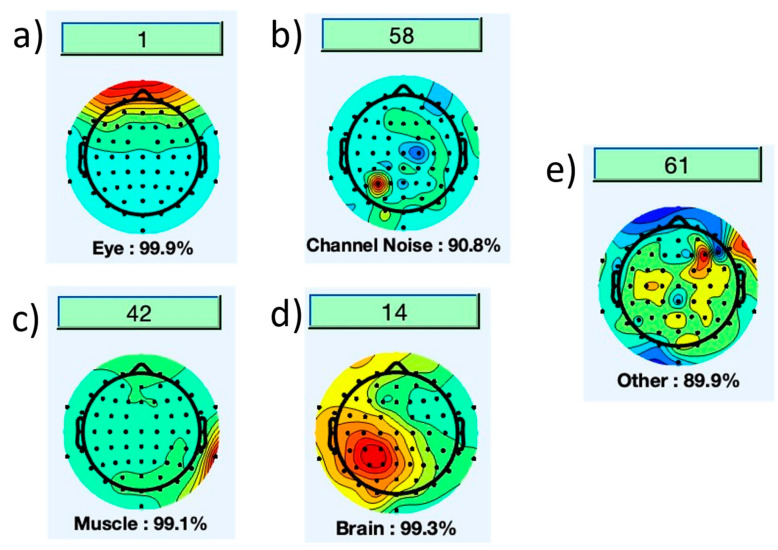
(**a**) channel 1 has 99.9% probability of eye blink, (**b**) channel 58 has 90.8% probability of channel linked noise, (**c**) channel 42 has 99.1% probability of artifact due to muscle activity, (**d**) channel 14 has 99.3% probability of brain signals, (**e**) channel 61 has 89.9% probability of other noise.

Independent component Analysis (ICA): Electroencephalography (EEG) signals are susceptible to artifacts caused by eye blinks, and muscle activity associated with movements of the head, nose, jaw, and tongue. Independent Component Analysis (ICA) was utilized to remove most of the artifact components from EEG signals. This statistical technique separates the artifacts into individual components, allowing for their identification and subsequent removal. The ICA technique was applied to the data through the EEGLab plugin. Initially, the data were decomposed by ICA, enabling the separation of the artifacts from the original brain signal in a channel-wise manner. The components (channels) were then classified into different labels such as eye, muscle, channel noise, etc., based on a certain percentage. The higher the percentage, the more likely that a particular label exists in that specific channel (Figure 2).

The percentage probability for removing the artifact or retaining the channel was set to 90, meaning that anything above 90% resulted in the removal of the artifact from that channel. Thus, implying only artifacts were removed while retaining the channel signal. This process is performed on all 64 channels. The threshold percentage can be varied, but 90% was found to be an optimal value. Determining the appropriate threshold is an essential parameter to consider, as the subsequent step involves removing the components of the artifacts.

**Figure 3:**
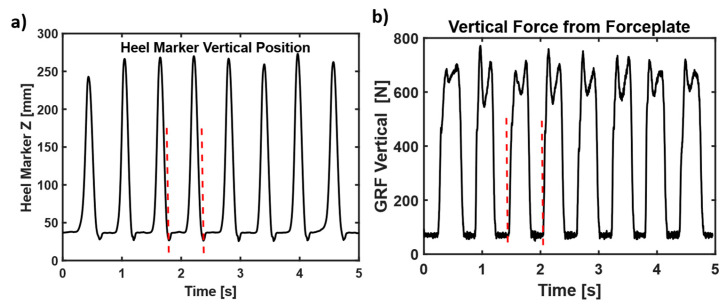
(**a**) A representative sample of heel vertical positions during walking. The heel strikes are the lowest position of heel marker during walking. (**b**) Forceplate vertical ground reaction forces. The sudden increase in vertical force is during heel strike. The red dashed lines represent a gait cycle.

Identification of Gait Cycles: Gait cycles events were identified during walking using the right and left side toe and heel markers. A representative graph showing heel contacts from (i) heel marker and (ii) forceplates is shown in Figure 3. The data from left and right side forceplates was used to confirm the gait events. A gait cycle is measured from any gait event (such as heel strike) to the same subsequent event on the same foot. While walking, the lowest vertical position of heel marker is during heel strike as shown in Figure 3a below and heel strike suddenly increases the vertical GRF as shown in Figure 3b. The red-dashed lines represent a gait cycle in representative graphs shown in figures below.

**Figure 4:**
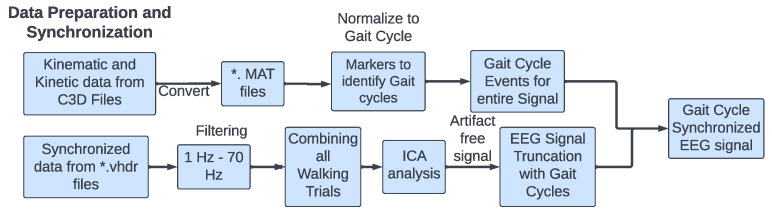
Schematic steps followed for EEG data preparation and synchronization with gait cycle events. Where ‘*’ refers to any file name for MAT files.

Extracting synchronized EEG data with Gait cycles: The gait events were used to truncate the EEG signals into individual gait cycles. Features were extracted from EEG signals during gait cycles. The overall data segmentation into gait cycles is summarized in Figure 4. The EEG data segmentation into gait cycles and procedures was inspired by similar signal analysis approaches [17,18].

**Figure 5:**
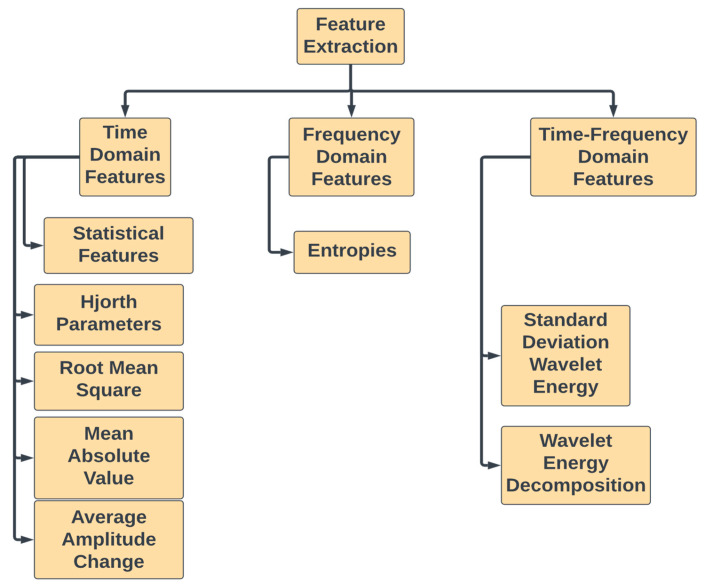
Features extracted from time domain, frequency domain, and time-frequency domain features.

EEG Feature extraction during gait cycles: One common approach for analyzing EEG signals is to extract features that can be used to classify different states or activities of the brain during different walking conditions. The features were extracted from normalized (0–100% gait cycle) segmented data from EEG. There are many different feature extraction techniques that can be applied to EEG signals such as time domain, frequency domain, and time-frequency features (Figure 5). The Hjorth parameters were calculated for both alpha and beta band signals across the EEG signals. The features of all channels corresponding to a single gait cycle sample are combined to create the initial feature vector.

Time domain features: The purpose of time domain features is to describe the behavior of a signal over a specific time interval. These features are properties of a signal that can be derived from its temporal characteristics. They are typically based on the shape of the signal waveform over time and can provide information about the signal’s frequency spectrum, amplitude, and phase. The ease of computation and interpretation of time domain features make them a valuable tool in signal analysis.

Mean Absolute Deviation (MAD): The mean absolute deviation (MAD) is a time domain feature that reflects the average distance between each data point and the mean. The computation of MAD involves taking the absolute value of each sample deviation from mean, summing them up, and dividing by the number of samples. The MAD is frequently utilized as an indicator of the signal’s strength or intensity, and can be useful in signal processing and machine learning applications. Due to its simplicity, the MAD is a widely adopted feature in various domains.

(1) $$MAV=\frac{1}{n}\sum_{i=1}^{n}\left|x_{i}-m(X)\right|$$

where m(X) is average value of the data set, n is number of data values. Since each EEG dataset is normalized to 100% of gait cycle, thus n = 100 for normalized gait cycles, and x_i_ is the data values in the set as shown in Equation (1).

Root Mean Square (RMS): The root mean square (RMS) is a time domain feature that quantifies the quadratic mean of a signal’s samples during a given time period. This feature is computed by squaring each sample within the interval, summing them up, and taking the square root of the sum. The RMS is commonly used as an indicator of the signal’s power or intensity and can be informative in signal analysis and machine learning tasks. The simplicity of the RMS computation makes it a popular feature in various fields.

(2) $$RMS=\sqrt{\frac{1}{n}\sum_{i}^{n}x_{i}^{2}}$$

where n is number of measurements (n = 100 for normalized gait cycles) and x_i_ is each value as shown in Equation (2). Similarly, statistical features like mean, variance, skewness, and kurtosis can be evaluated.

Hjorth parameters: Hjorth parameters are a set of time-domain measures used to characterize the activity of a signal, typically an electroencephalogram (EEG) signal or other biological signals such as electromyography (EMG) or electrocardiography (ECG) signals. The three Hjorth parameters are:

1. Activity: It is a measure of the total power of a signal, obtained by calculating the variance of the signal as shown in Equation (3) below. This parameter provides an overall measure of the signal’s intensity or activity level.
  (3) $$Activity=var(y\left(t\right))$$
2. Mobility: It is a measure of the frequency content of the signal, obtained by calculating the standard deviation of the first derivative of the signal as shown in Equation (4) below. This parameter provides an indication of how much the signal changes over time and is related to the signal’s frequency or “mobility”.
  (4) $$Mobility=\sqrt{\frac{var(y^{\prime }\left(t\right))}{var(y\left(t\right))}}$$
3. Complexity: It is a measure of the irregularity or complexity of the signal, obtained by calculating the standard deviation of the second derivative of the signal as shown in Equation (5) below. This parameter provides an indication of how much the signal deviates from a smooth or regular waveform, and is related to the signal’s complexity.
  (5) $$Complexity=\frac{mobility(y^{\prime }\left(t\right))}{mobility(y\left(t\right))}$$

Frequency domain features: These features are based on the spectral content of the EEG signal. They can be computed using Fourier transform or wavelet analysis. Examples include power spectral density, frequency band power ratios, and peak frequency.

Shannon Entropy: Shannon Entropy is a measure of the amount of uncertainty or randomness in a system. In the context of EEG signals, Shannon Entropy has been used as a measure of the complexity or diversity of neural activity. It is computed as shown in Equation (6) below.

(6) $$H\left(X\right)=-\sum_{i}P\left(x_{i}\right)\log\;P(x_{i})$$

Time-frequency features: These features combine time-domain and frequency-domain information by analyzing changes in frequency content over time. Examples include time-frequency power spectrograms, wavelet-based scalograms, and spectrogram-based features.

Features from Wavelet Analysis: Wavelet analysis can be a powerful tool for analyzing EEG signals, as it allows for the extraction of features and the identification of specific frequency bands associated with different brain activities or events. In wavelet analysis, a wavelet function is used to decompose the signal into different scales, where each scale represents a different frequency band. The decomposition process generates a set of coefficients, which can be used to reconstruct the original signal. The coefficients can also be used to extract information about the signal, such as the energy in each frequency band. In EEG signal analysis, the energy in the detail coefficients can be used to identify specific frequency bands that are associated with certain brain activities or events. For example, the energy in the beta frequency band (12–30 Hz) in the detail coefficients may be used to identify changes in brain activity associated with motor movement or cognitive processing. Similarly, the energy in the alpha frequency band (8–12 Hz) in the detail coefficients may be used to identify changes in brain activity associated with relaxation or meditation. SD of Wavelet Energy: The standard deviation of wavelet energy refers to the statistical measure that describes the amount of variation or dispersion in the energy values of a wavelet transform. In signal processing, the wavelet transform is used to analyze signals at different scales, and the energy of the wavelet coefficients represents the strength of the signal at each scale. The standard deviation of wavelet energy is used to characterize the variability of the energy across different scales.

(7) $$SD\;of\;wavelet\;energy=\sqrt{\frac{1}{N}\sum_{i}^{N}({E\left(i\right)-mean(E))}^{2}}$$

where N is the total number of wavelet coefficients, E(i) is the energy of the ith coefficient, and mean (E) is the mean energy value as shown in Equation (7) above.

DWT is particularly suitable for representing non-stationary signals, such as EEG signals, because it provides optimal resolution in both time and frequency domains. To extract the features from each EEG signal, denoted as X(z), a decomposition process is performed using DWT to obtain detailed and approximate coefficients across various frequency bands. This process involves dividing the EEG signal into two bands using a high-pass filter and a low-pass filter, until the desired level of decomposition is reached. The detailed coefficients are derived by filtering the signal using a high-pass filter (H(z)), while the approximations are obtained using a low-pass filter (L(z)). To ensure a reliable analysis of EEG signals using DWT, the number of decompositions and the wavelet function employed must be carefully considered. In this study, we have utilized a four-level decomposition and the Daubechies-2 wavelet function. The Daubechies-2 wavelet function is well-suited for EEG analysis due to its similarity to the spike-wave pattern found in EEG, and its scalability and flexibility for addressing boundary problems [19]. Additionally, we have chosen four-level decomposition as it effectively captures the dominant frequency bands present in EEG.

**Figure 6:**
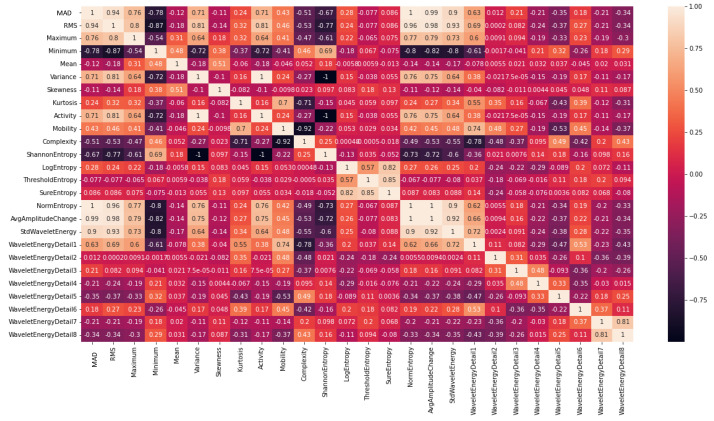
Correlation matrix of features (MAD, RMS, maximum, minimum, mean, variance, skewness, kurtosis, Activity, Mobility, Complexity, Shannon Entropy, Log Entropy, Threshold Entropy, Sure Entropy, Norm Entropy, Average amplitude change, standard deviation of wavelet energy, wavelet energy for detail 1–8) used as input for ML algorithms.

Feature selection: The feature set included MAD, RMS, maximum, minimum, mean, variance, skewness, kurtosis, Activity, Mobility, Complexity, Shannon Entropy, Log Entropy, Threshold Entropy, Sure Entropy, Norm Entropy, Average amplitude change, standard deviation of wavelet energy, wavelet energy for detail 1–8. The correlation matrix of features are shown in Figure 6 below. Due to the high number of features in comparison to the number of samples, there is a significant likelihood of having redundant and noisy features in the feature set. To address this issue, a feature selection method was implemented in this study. The absolute value of the standardized u-statistic of a two-sample unpaired Wilcoxon test (also known as the Mann–Whitney test) [20] was selected as the criterion to identify distinctive and informative features. Additionally, the average of the absolute values of the cross-correlation coefficient between a candidate feature and all previously selected features was calculated to further reduce the number of features. Features that were highly correlated with the features already selected were less likely to be included in the output list. This procedure resulted in a reduced and more distinctive set of features for successful classification.

Classification Algorithms: The ultimate goal of this study was to evaluate classification performance of ML methods for three different cases: (i) four classes (normal walking, normal walking with Stroop task, normal walking with medio-lateral perturbation (MLP), and normal walking with COM visual feedback (COM)), (ii) three classes (normal walking, normal walking with Stroop task, and normal walking with COM visual feedback), (iii) two classes (stable versus unstable gait). The rationale was to not only classify two conditions (stable versus unstable) as usually assessed in clinical practice but also distinguish subtle gait changes induced due to (i) dual-tasking like Stroop, (ii) stabilization of gait due to visual COM feedback, and (iii) MLP. Stable gait was defined as normal walking and walking with COM visual feedback. Similarly, unstable gait was defined as walking with Stroop, and walking with the medio-lateral perturbation condition (MLP). For real-time applications, the classifiers require high sensitivity and high specificity to meet challenging instability situations during gait.

Support Vector Machines (SVM): With high sensitivity and specificity in mind, we tested SVM classifier [21,22] with RBF kernel. Five-fold cross-validation was used to evaluate the performance of the classifiers. In each classification step, one-fold was designated as the test set while the remaining folds were used to train the classifier. Each fold was used only once as the test set, and the performance metrics were averaged across all the folds.

**Figure 7:**
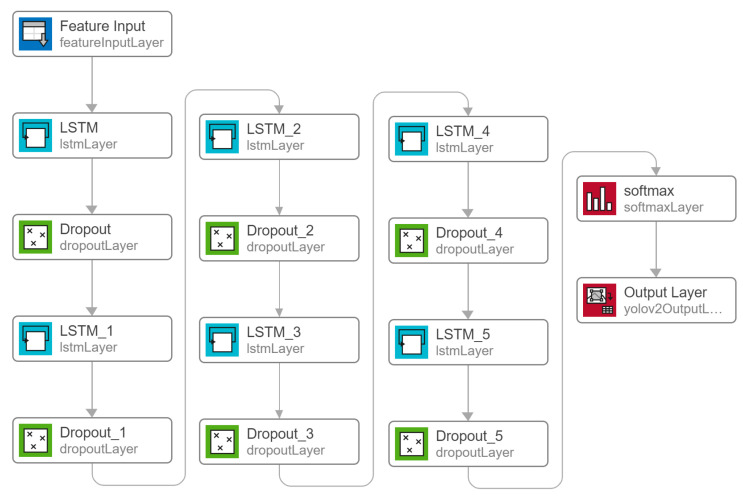
RNN model developed with multiple layers of LSTM and dropout layers.

Recurrent Neural Network (Figure 7): A Recurrent Neural Network (RNN) is a type of artificial neural network (ANN) that is commonly used in the processing of sequential data. RNNs are capable of processing inputs of variable length, where each input is dependent on the previous ones, making them useful for tasks such as language modeling, speech recognition, and time series analysis. RNNs can process the temporal dynamics of EEG signals in a time series format, which is essential for the detection of epileptiform discharges, which are abnormal electrical discharges in the brain that are associated with epilepsy [23]. By processing the EEG signals in a time series format, RNNs can capture the sequential dependencies between the different time points of the EEG signal and identify patterns that may be indicative of gait instability. Previous studies have reported that RNNs can outperform traditional machine learning methods, for example, the ChronoNet model, which is a deep RNN, was specifically designed for identifying epileptiform discharges in EEG signals and has shown high accuracy in detecting these abnormal patterns [23]. There are several variations of RNNs, such as Long Short-Term Memory (LSTM) and Gated Recurrent Unit (GRU), which were designed to address some of the limitations of standard RNNs, such as the vanishing gradient problem. LSTM and GRU networks use gating mechanisms to control the flow of information through the network and have been shown to be more effective in handling long-term dependencies in sequential data.

Long Short-Term Memory (LSTM) represents a more sophisticated version of Recurrent Neural Networks (RNNs) that integrates three gates to regulate the retention and transmission of information, thereby resolving the challenge of the vanishing gradient that often occurs during regular RNN training [24]. In addition to the common parameters used in Temporal Convolutional Networks (TCNs), the optimization of Recurrent Neural Networks such as LSTM and GRU encompassed various factors such as the number of hidden units and layers, standard deviation for layer initialization, and clipping strength, which aims to prevent the gradient from exploding.

**Figure 8:**
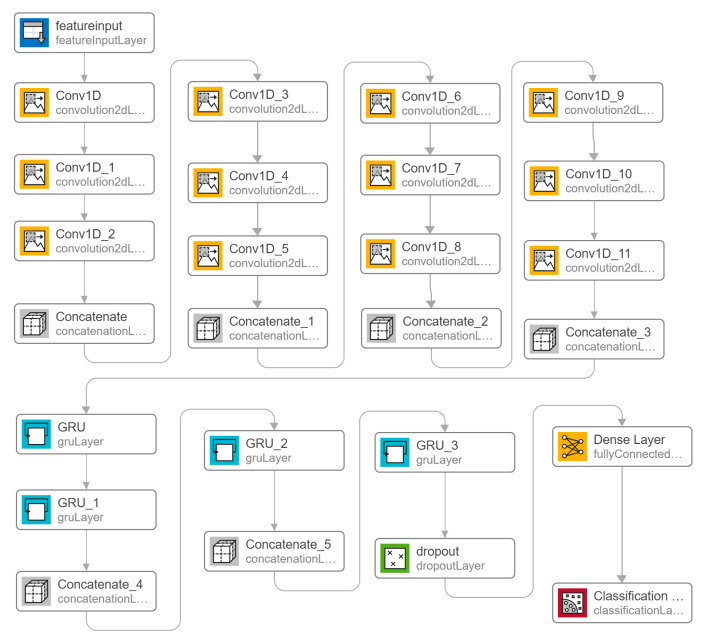
ChronoNet developed with multiple layers of 1D convolutional layers, GRU, and dropout layer.

ChronoNet (Figure 8): A deep recurrent neural network (RNN) called ChronoNet, can be designed to identify abnormal patterns in electroencephalogram (EEG) signals [23]. The ChronoNet model processes raw EEG data in a time series format, allowing it to capture the temporal dynamics and patterns of EEG signals over time. The architecture of ChronoNet involves the arrangement of several 1D convolution layers and deep gated recurrent unit (GRU) layers. Each 1D convolution layer utilizes numerous filters with lengths that increase exponentially. The stacked GRU layers are densely connected in a feed-forward manner.

Extreme Gradient Boosting (XGBoost) is a machine learning algorithm that utilizes a supervised learning approach to accurately predict an objective variable by amalgamating the predictions of several weaker models. This algorithm is a widely used data mining tool that exhibits good speed and performance. Compared to the Random Forest model, the XGBoost model can generate predictions at a speed that is 10-times faster. The XGBoost model is developed using the additive tree method, where a new tree is added at each step to augment the existing trees that have been constructed. As more trees are added, the model’s accuracy generally improves.

## 3. Results

Participants walked in four different conditions as shown in Figure 1. Prediction of stable and unstable walking conditions: To evaluate how different ML algorithms perform with the same processing pipeline and feature sets as inputs, we performed a rigorous comparison of all ML methods. The accuracy and loss were used as evaluation criteria in the EEG pattern recognition for: (i) four classes (normal walking, normal walking with Stroop task, normal walking with medio-lateral perturbation, and normal walking with COM visual feedback), (ii) three classes (normal walking, normal walking with Stroop task, and normal walking with COM visual feedback), and (iii) two classes (stable versus unstable gait). Accuracy sensitivity and specificity showed performance of classifiers in predicting all i–iii cases. The best classification accuracy for four classes (normal walking, Stroop, COM and MLP) was achieved by ChronoNet (68.0%), followed by XG Boosting (67.6%), Random Forest (67.3%), RNN (67.2%), and SVM (59.7%) (Table 1). The best classification accuracy for three classes (normal walking, Stroop, and COM) was achieved by ChronoNet (77.4%), followed by XG Boosting (76.8%), RNN (76.4%), Random Forest (75.8%), and SVM (69.4%) (Table 2). The best classification accuracy for two classes (stable and unstable) was achieved by RNN (82.0%), followed by ChronoNet (79.6%), Random Forest (79.2%), XG Boosting (78.4%), and SVM (73.8%) (Table 3).

**Figure 9:**
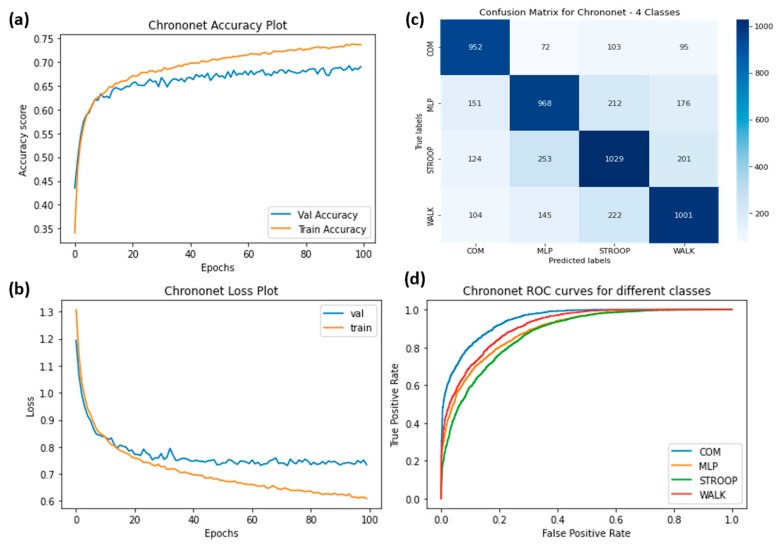
(**a**) Training accuracy of ChronoNet for four classes (normal walking, Stroop, COM and MLP), (**b**) training losses for the four classes, (**c**) confusion matrix, and (**d**) ROC curves for all four classes.

**Figure 10:**
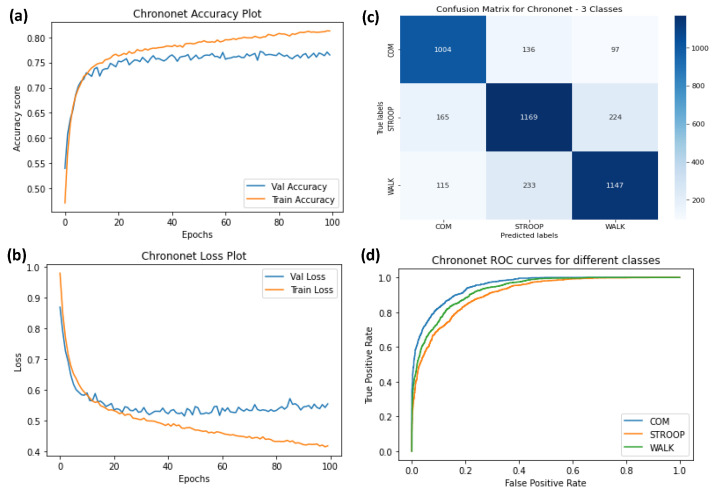
(**a**) Training accuracy of ChronoNet for three classes (normal walking, Stroop, and COM), (**b**) training losses for the three classes, (**c**) confusion matrix, and (**d**) ROC curves for all three classes.

**Figure 11:**
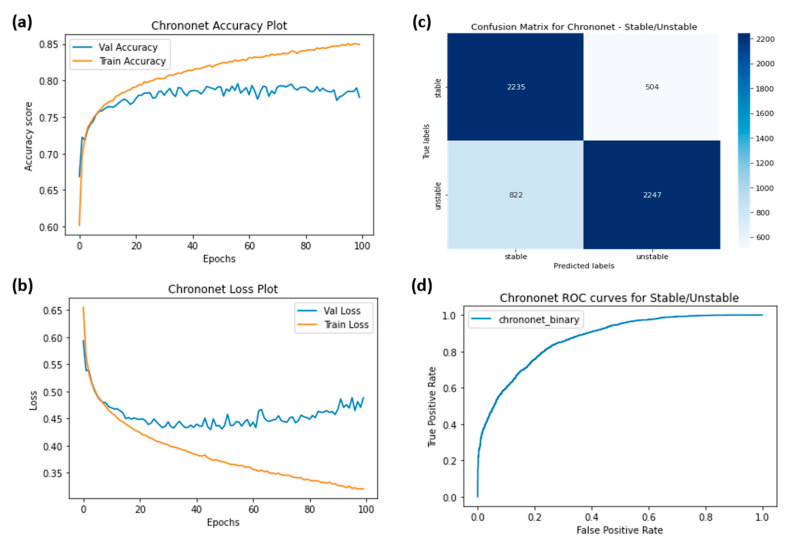
(**a**) Training accuracy of ChronoNet for two classes (stable and unstable), (**b**) training losses for the two classes, (**c**) confusion matrix, and (**d**) ROC curves for the two classes.

**Figure 12:**
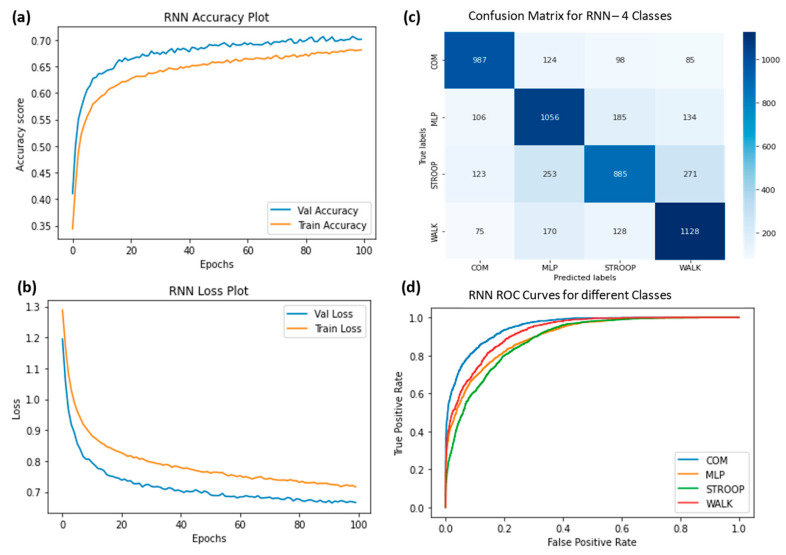
(**a**) Training accuracy of RNN for four classes (normal walking, Stroop, COM and MLP), (**b**) training losses for the four classes, (**c**) confusion matrix, and (**d**) ROC curves for all four classes.

**Figure 13:**
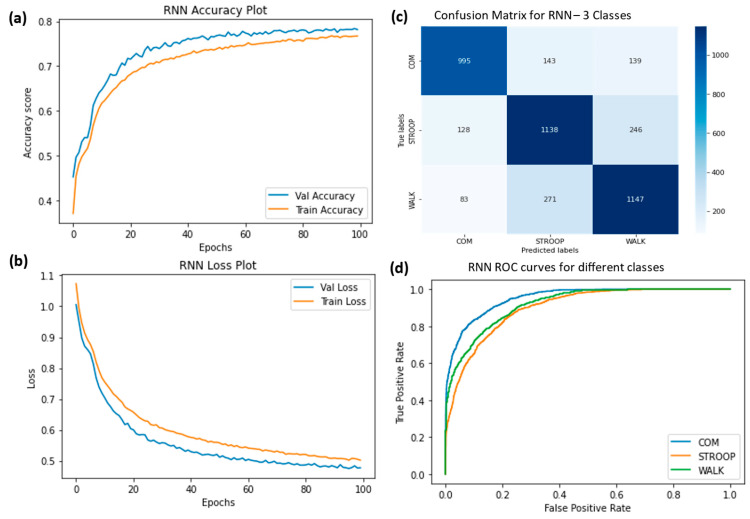
(**a**) Training accuracy of RNN for three classes (normal walking, Stroop, and COM), (**b**) training losses for the three classes, (**c**) confusion matrix, and (**d**) ROC curves for all three classes.

**Figure 14:**
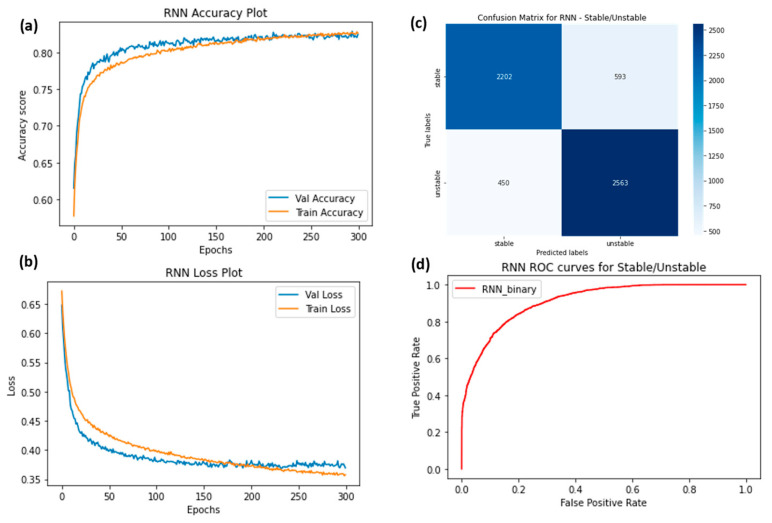
(**a**) Training accuracy of RNN for two classes (stable and unstable), (**b**) training losses for the two classes, (**c**) confusion matrix, and (**d**) ROC curves for the two classes.

The ROC curves were plotted for ChronoNet for four classes (Figure 9), ChronoNet for three classes (Figure 10), ChronoNet for two classes (Figure 11), RNN for four classes (Figure 12), RNN for two classes (Figure 13), and RNN for one classes (Figure 14).

## 4. Discussion

Advancements in technology have made it possible to monitor brain activity while a person is walking in real-time. In particular, non-invasive and mobile EEG or BCI recording caps are used to monitor cortical activity during actual gait. The use of EEG signals and machine learning to detect gait instability is an emerging field of research. Further research is needed to develop more accurate and reliable algorithms for detecting gait instability from EEG signals. However, the potential benefits of this technology are significant, and it is likely to play an increasingly important role in the diagnosis and intervention of gait instability. The main objective of this study was to investigate ML algorithms and their performance on gait stability classification using EEG signals during walking. In this study, participants walked under various conditions, including (i) normal walking, (ii) normal walking with Stroop task, (iii) normal walking with COM visual feedback, and (iv) normal walking with medio-lateral perturbations (MLP). We grouped conditions like normal walking and walking with COM visual feedback as stable walking conditions, while walking with medio-lateral perturbations and dual-tasking (like Stroop) as less stable (or unstable) conditions. Support Vector Machines (SVM), Random Forest, XG Boosting, Recurrent Neural Network (RNN), and ChronoNet were employed for the classification of gait type and stability.

Undoubtedly, BCIs have the potential to revolutionize the field of assistive technology, providing new opportunities for people with disabilities to interact with the world around them and improve their gait and quality of life. Some of the potential benefits of using EEG signals and machine learning to detect gait instability are: (i) non-invasive signals that are relatively inexpensive to obtain, (ii) automatic detection of gait instability, (iii) automaticity will save time and resources, (iv) advantageous in continuous monitoring of gait instability over time, and (v) facilitation of personalized treatments for gait instability. In order to improve the use of EEG signals and machine learning to detect gait instability, the accuracy of machine learning algorithms needs to be established. We found the best classification accuracy for two classes (stable and unstable) was achieved by RNN (82.0%), followed by ChronoNet (79.6%) and Random Forest (79.2%). However, these classification accuracies decreased if classification labels (classes) increased to three and four. We found the best classification accuracy for three classes (normal walking, Stroop, and COM) was achieved by ChronoNet (77.4%), followed by XG Boosting (76.8%) and RNN (76.4%). The best classification accuracy for four classes (normal walking, Stroop, COM, and MLP) was achieved by ChronoNet (68.0%) followed by XG Boosting (67.6%) and Random Forest (67.3%). Currently, the model is developed on controlled laboratory data and the algorithms need to be able to detect gait instability in a variety of conditions outside laboratory. We acknowledge that the classification accuracies are quite low even when using such state-of-the-art ML algorithms. This may be due to the fact that walking stability under varying conditions is not only dependent on brain mechanisms but also on multiple interaction of factors such as aging, weight, gender, and health status (healthy versus frail). Despite the challenges, the potential benefits of using EEG signals and machine learning to detect gait instability are significant. This technology has the potential to improve the diagnosis and treatment of gait instability, and it is likely to play an increasingly important role in the future.

In this study, Independent Component Analysis (ICA) was used in conjunction with other filtering algorithms to remove artifacts from EEG data. However, the computational complexity associated with EEG source analysis, particularly when combining ICA with blind source localization, can be significant. As a result, many ICA-based EEG analysis tools are designed for offline processing. This work serves as a foundation for further future research in the area of developing quick real-time (or online) algorithms in the future. Nevertheless, there have been recent advancements in real-time EEG source mapping toolboxes, such as REST [25] and Online Recursive ICA (ORICA) [26], which utilize recursive Independent Component Analysis (ICA) [27] to provide a solution for the source separation problem in near real-time. These advancements enable low-latency access to source information. Therefore, the recent advancements in technology show potential for enabling innovations in experimental designs for many BCI systems. Additionally, traditional spatial filtering techniques, such as Laplacian filtering and common average referencing, are also available as alternatives [28,29]. These filters are designed to minimize the contribution of other EEG electrodes to each channel, thereby improving the isolation of information from each individual electrode. Such spatial filtering techniques can be useful alternatives until a more robust and reliable online ICA algorithm is available for real-time applications.

We used varying mechanical and cognitive constraints to influence the stability of gait condition during this study. To increase gait stability, we provided participants with visual feedback of COM. This may reduce translational variability while walking on the treadmill and result in more precise control maintaining a central position (or stability) on the treadmill [30]. Studies in populations without neurological injuries have shown that controlling the medio-lateral motion of the center of mass is important for maintaining dynamic balance and creating walking stability during directional changes [31,32]. Visual feedback of COM helps in maintaining slower COM motions that are believed to enhance gait stability and reducing perturbations [33]. In this study, participants were provided visual feedback of COM for stable gait.

To reduce gait stability, we used a dual task paradigm and also a mechanical perturbation. Walking is a task that requires significant attention and is often carried out while being distracted by other pedestrians, talking, or texting. To create a cognitive demanding task, a dual-task paradigm was utilized in this study, where participants were required to walk at the same time as performing the Stroop task [34]. During dual-task walking, both walking and cognitive task performance are negatively impacted, resulting in increased gait instability [35]. The walking stability is compromised since participants have to adaptively be able to shift their resources from the cognitive to the motor task, particularly when balance demands change, to protect their balance and avoid falling. The prioritization of motor or cognitive task performance during dual-task walking can be attributed to increased risk of falls. Low attention and cognition capabilities have also been linked to slow gait and poor stability [36,37]. Studies [38,39] have utilized dual-task strategies to assess the gait stability and variability of elderly individuals. Walking stability was also decreased in this study with the mediolateral treadmill perturbation.

We applied the treadmill perturbations at right heel strike of each stride. Since we applied MLP for each stride, the extent of the perturbations were adjusted as to avoid the need for recovery steps. MLP has been earlier reported to reduce gait stability [40].

Falls among older adults are often linked to gait instability and age-related changes can make it more difficult for older adults to maintain their balance and avoid falls. Thus, identifying and improving gait stability can help reduce the risk of falls. It is important for healthcare providers to utilize non-invasive portable technologies which accurately assess gait stability among older adults and develop individualized plans to reduce the risk of falls.

Limitations: There are some limitations to this study. All participant data were collected in controlled virtual reality environments while walking on treadmill for a limited duration. Additional research is needed to understand how fatigue during prolonged walking, cognitive distraction, and real-world environments affect stability and classification by ML models. The mobile EEG Brian Vision system was selected since this system is designed to remove movement artifacts. EEG artifacts can interfere with accurate interpretation of the EEG signal and may require preprocessing techniques to remove or minimize their effects. We adopted ICA and filtering for the artifact removal procedure. ML methods are designed to be robust to noise and are helpful in identifying specific patterns in noisy EEG signals. We applied feature engineering techniques to identify the most relevant features in the data and exclude features that are more likely to be noise. This helped to improve the accuracy of the classification algorithm by reducing the amount of noise in the data. For example, decision tree algorithms are known to be relatively insensitive to noise in the data.

## 5. Conclusions

EEG signals offer great promise as a tool for evaluating and monitoring walking stability. We have successfully demonstrated that ChronoNet, RNN, and XG Boosting models can accurately classify stable gait. The utilization of head-worn wearables or brain-computer interfaces (BCIs) holds potential for the real-time assessment of gait stability. This study presents a fundamental investigation into classification models that could have significant value in the assessment of gait stability among older adults. Furthermore, this research serves as a basis for further exploration of techniques aimed at real-time analysis.

## Figures and Tables

**Table 1:** Performance score of different algorithms for four classes (normal walking, Stroop, COM and MLP).

Models	Accuracy	Precision	Recall	F-Score	MCC	ROC Area	AUC
SVM	0.5977	0.5991	0.6021	0.5989	0.4646	0.8376	0.5667
Random Forest	0.6736	0.6752	0.6756	0.6753	0.5642	0.8820	0.5667
XG Boosting	0.6764	0.6787	0.6782	0.6783	0.5680	0.8968	0.5841
RNN	0.6728	0.6729	0.6729	0.6729	0.5638	0.8961	0.6070
ChronoNet	0.6800	0.6812	0.6854	0.6828	0.5729	0.9036	0.5901

**Table 2:** Performance score of different algorithms for three classes (normal walking, Stroop, and COM).

Models	Accuracy	Precision	Recall	F-Score	MCC	ROC Area	AUC
SVM	0.6944	0.6950	0.6998	0.6951	0.5438	0.8627	0.8372
Random Forest	0.7588	0.7594	0.7602	0.7597	0.6371	0.9054	0.8372
XG Boosting	0.7685	0.7698	0.7701	0.7834	0.5687	0.7827	0.5841
RNN	0.7645	0.7689	0.7653	0.7718	0.5983	0.9166	0.5991
ChronoNet	0.7744	0.7744	0.7763	0.7752	0.6596	0.9295	0.8408

**Table 3:** Performance score of different algorithms for stable and unstable.

Models	Accuracy	Precision	Recall	F-Score	MCC	ROC Area	AUC
SVM	0.7388	0.7413	0.7356	0.7360	0.4770	0.7908	0.7908
Random Forest	0.7919	0.7918	0.7908	0.7911	0.5826	0.7908	0.7908
XG Boosting	0.7848	0.7859	0.7827	0.7834	0.5687	0.7827	0.7827
RNN	0.8204	0.8212	0.8192	0.8197	0.6404	0.8192	0.8192
ChronoNet	0.7963	0.7967	0.7957	0.7959	0.5924	0.7957	0.7957

## Data Availability

The raw data supporting the conclusions of this article will be made available by the corresponding author upon reasonable request.

## References

[1] Moreland B., Kakara R., Henry A. (2020). Trends in Nonfatal Falls and Fall-Related Injuries Among Adults Aged ≥65 Years—United States, 2012–2018. MMWR Morb. Mortal. Wkly. Rep..

[2] Parkkari J., Kannus P., Palvanen M., Natri A., Vainio J., Aho H., Vuori I., Järvinen M. (1999). Majority of Hip Fractures Occur as a Result of a Fall and Impact on the Greater Trochanter of the Femur: A Prospective Controlled Hip Fracture Study with 206 Consecutive Patients. Calcif. Tissue Int..

[3] Hayes W.C., Myers E.R., Morris J.N., Gerhart T.N., Yett H.S., Lipsitz L.A. (1993). Impact near the hip dominates fracture risk in elderly nursing home residents who fall. Calcif. Tissue Int..

[4] Tinetti M.E., Kumar C. (2010). The Patient Who Falls. JAMA.

[5] Tinetti M.E. (2003). Preventing Falls in Elderly Persons. N. Engl. J. Med..

[6] Hamacher D., Singh N.B., Van Dieën J.H., Heller M.O., Taylor W.R. (2011). Kinematic measures for assessing gait stability in elderly individuals: A systematic review. J. R. Soc. Interface.

[7] Bruijn S.M., Meijer O.G., Beek P.J., van Dieën J.H. (2013). Assessing the stability of human locomotion: A review of current measures. J. R. Soc. Interface.

[8] Morris R., Lord S., Bunce J., Burn D., Rochester L. (2016). Gait and cognition: Mapping the global and discrete relationships in ageing and neurodegenerative disease. Neurosci. Biobehav. Rev..

[9] Fukuyama H., Ouchi Y., Matsuzaki S., Nagahama Y., Yamauchi H., Ogawa M., Kimura J., Shibasaki H. (1997). Brain functional activity during gait in normal subjects: A SPECT study. Neurosci. Lett..

[10] Bruijn S.M., Van Impe A., Duysens J., Swinnen S.P. (2014). White matter microstructural organization and gait stability in older adults. Front. Aging Neurosci..

[11] Jahn K., Deutschländer A., Stephan T., Strupp M., Wiesmann M., Brandt T. (2004). Brain activation patterns during imagined stance and locomotion in functional magnetic resonance imaging. NeuroImage.

[12] Snijders A.H., Leunissen I., Bakker M., Overeem S., Helmich R.C., Bloem B.R., Toni I. (2011). Gait-related cerebral alterations in patients with Parkinson’s disease with freezing of gait. Brain.

[13] Wang C., Wai Y., Kuo B., Yeh Y.-Y., Wang J. (2008). Cortical control of gait in healthy humans: An fMRI study. J. Neural Transm..

[14] Harada T., Miyai I., Suzuki M., Kubota K. (2008). Gait capacity affects cortical activation patterns related to speed control in the elderly. Exp. Brain Res..

[15] Jahn K., Deutschländer A., Stephan T., Kalla R., Wiesmann M., Strupp M., Brandt T. (2008). Imaging human supraspinal locomotor centers in brainstem and cerebellum. NeuroImage.

[16] Sipp A.R., Gwin J.T., Makeig S., Ferris D.P. (2013). Loss of balance during balance beam walking elicits a multifocal theta band electrocortical response. J. Neurophysiol..

[17] Zhang J., Lockhart T.E., Soangra R. (2014). Classifying lower extremity muscle fatigue during walking using machine learning and inertial sensors. Ann. Biomed. Eng..

[18] Soangra R., Wen Y., Yang H., Grant-Beuttler M. (2022). Classifying Toe Walking Gait Patterns Among Children Diagnosed with Idiopathic Toe Walking Using Wearable Sensors and Machine Learning Algorithms. IEEE Access.

[19] Lema-Condo E.L., Bueno-Palomeque F.L., Castro-Villalobos S.E., Ordonez-Morales E.F., Serpa-Andrade L.J. Comparison of wavelet transform symlets (2-10) and daubechies (2-10) for an electroencephalographic signal analysis. Proceedings of the 2017 IEEE XXIV International Conference on Electronics, Electrical Engineering and Computing (INTERCON).

[20] Kruskal W.H. (1957). Historical Notes on the Wilcoxon Unpaired Two-Sample Test. J. Am. Stat. Assoc..

[21] Antony M.J., Sankaralingam B.P., Mahendran R.K., Gardezi A.A., Shafiq M., Choi J.G., Hamam H. (2022). Classification of EEG Using Adaptive SVM Classifier with CSP and Online Recursive Independent Component Analysis. Sensors.

[22] Ganaie M.A., Tanveer M., Jangir J. (2022). EEG signal classification via pinball universum twin support vector machine. Ann. Oper. Res..

[23] Roy S., Kiral-Kornek I., Harrer S. (2019). ChronoNet: A Deep Recurrent Neural Network for Abnormal EEG Identification. Artif. Intell. Med..

[24] Tortora S., Ghidoni S., Chisari C., Micera S., Artoni F. (2020). Deep learning-based BCI for gait decoding from EEG with LSTM recurrent neural network. J. Neural Eng..

[25] Pion-Tonachini L., Hsu S.H., Makeig S., Jung T.P., Cauwenberghs G. (2015). Real-time EEG Source-mapping Toolbox (REST): Online ICA and source localization. Annu. Int. Conf. IEEE Eng. Med. Biol. Soc..

[26] Hsu S.H., Mullen T., Jung T.P., Cauwenberghs G. (2014). Online recursive independent component analysis for real-time source separation of high-density EEG. Annu. Int. Conf. IEEE Eng. Med. Biol. Soc..

[27] Akhtar M.T., Jung T.-P., Makeig S., Cauwenberghs G. Recursive independent component analysis for online blind source separation. Proceedings of the 2012 IEEE International Symposium on Circuits and Systems.

[28] Hortal E., Ubeda A., Ianez E., Azorin J.M., Fernandez E. (2016). EEG-Based Detection of Starting and Stopping During Gait Cycle. Int. J. Neural Syst..

[29] Ortiz M., Rodriguez-Ugarte M., Ianez E., Azorin J.M. (2017). Application of the Stockwell Transform to Electroencephalographic Signal Analysis during Gait Cycle. Front. Neurosci..

[30] Anson E., Rosenberg R., Agada P., Kiemel T., Jeka J. (2013). Does visual feedback during walking result in similar improvements in trunk control for young and older healthy adults?. J. Neuroeng. Rehabil..

[31] Schrager M.A., Kelly V.E., Price R., Ferrucci L., Shumway-Cook A. (2008). The effects of age on medio-lateral stability during normal and narrow base walking. Gait Posture.

[32] Patla A.E., Adkin A., Ballard T. (1999). Online steering: Coordination and control of body center of mass, head and body reorientation. Exp. Brain. Res..

[33] Tesio L., Rota V. (2019). The Motion of Body Center of Mass During Walking: A Review Oriented to Clinical Applications. Front. Neurol..

[34] Woollacott M., Shumway-Cook A. (2002). Attention and the control of posture and gait: A review of an emerging area of research. Gait Posture.

[35] Beurskens R., Bock O. (2012). Age-related deficits of dual-task walking: A review. Neural Plast..

[36] Martin K.L., Blizzard L., Wood A.G., Srikanth V., Thomson R., Sanders L.M., Callisaya M.L. (2013). Cognitive Function, Gait, and Gait Variability in Older People: A Population-Based Study. J. Gerontol. Ser. A.

[37] Mielke M.M., Roberts R.O., Savica R., Cha R., Drubach D.I., Christianson T., Pankratz V.S., Geda Y.E., Machulda M.M., Ivnik R.J. (2012). Assessing the Temporal Relationship Between Cognition and Gait: Slow Gait Predicts Cognitive Decline in the Mayo Clinic Study of Aging. J. Gerontol. Ser. A Biol. Sci. Med. Sci..

[38] Parker T.M., Osternig L.R., Lee H.J., Donkelaar P., Chou L.S. (2005). The effect of divided attention on gait stability following concussion. Clin. Biomech. (Bristol. Avon.).

[39] Springer S., Giladi N., Peretz C., Yogev G., Simon E.S., Hausdorff J.M. (2006). Dual-tasking effects on gait variability: The role of aging, falls, and executive function. Mov. Disord..

[40] Li J., Huang H.J. (2022). Small directional treadmill perturbations induce differential gait stability adaptation. J. Neurophysiol..

